# Supplementary material for: Direct observation of a few-photon phase shift induced by a single quantum emitter in a waveguide
Source: Nat Commun. 2024 Aug 31;15:7583. doi: 10.1038/s41467-024-51805-9 (PMC11365957; doi:10.1038/s41467-024-51805-9)
Supplement: Supplementary file 1 — Supplementary Information [file 41467_2024_51805_MOESM1_ESM.pdf]

# Supplemental Material

Mathias J.R. Staunstrup,[1] Alexey Tiranov,[1] Ying Wang,[1] Sven Scholz,[2] Andreas D. Wieck,[2] Arne Ludwig,[2] Leonardo Midolo,[1] Nir Rotenberg,[1] Peter Lodahl,[1,*] and Hanna Le Jeannic[1,†]

[1]*Center for Hybrid Quantum Networks (Hy-Q), Niels Bohr Institute, University of Copenhagen, DK-2100 Copenhagen Ø, Denmark*

[2]*Lehrstuhl für Angewandte Festkörperphysik, Ruhr-Universität Bochum, Universitätsstraße 150, 44801 Bochum, Germany*

## I. TRANSMISSION OF THE EMITTER-WAVEGUIDE SYSTEM

The quantum dot is modeled as a two-level system (TLS) with ground-and excited states $|g\rangle$ and $|e\rangle$. The Hamiltonian describing the light-emitter interaction can be written as[1]:

$$\hat{H} = -\hbar\Delta\hat{\sigma}_{eg}\hat{\sigma}_{ge} + \hbar\omega_p\hat{\mathbf{f}}^\dagger(\mathbf{r})\hat{\mathbf{f}}(\mathbf{r}) - \hat{\mathbf{d}}\cdot\hat{\mathbf{E}}(\mathbf{r}) \tag{1}$$

The first term describes the emitter dynamics with $\Delta = \omega - \omega_{\mathrm{TLS}}$ as the detuning between the driving field of frequency $\omega$ and the two-level system resonance $\omega_{\mathrm{TLS}}$. $\hat{\sigma}_{ij} = |i\rangle\langle j|$, where $i, j \in \{|g\rangle, |e\rangle\}$ are the transition operators of the TLS. The second term in the Hamiltonian accounts for the photon field at position $\mathbf{r}$ with the bosonic annihilation operators $\hat{\mathbf{f}}(\mathbf{r})$. Finally, the last term accounts for the light-matter interaction between the emitter dipole $\hat{\mathbf{d}}$ and the electric field $\hat{\mathbf{E}}(\mathbf{r}) = \hat{\mathbf{E}}^+(\mathbf{r}) + \hat{\mathbf{E}}^-(\mathbf{r})$. The response of the TLS can be expressed by the partially traced density matrix giving the elements $\rho_{ij}$. In the rotating wave approximation and solving for the steady state solution ($\dot{\rho} = 0$) we obtain the elements:

$$\begin{aligned}\rho_{ee} &= \frac{2\gamma_2\Omega^2}{\gamma(\gamma_2^2 + \Delta^2 + 4(\gamma_2/\gamma)\Omega^2)}\\ \rho_{ge} &= -\frac{\Omega(i\gamma_2 + \Delta)}{\gamma_2^2 + \Delta^2 + 4(\gamma_2/\gamma)\Omega^2}\end{aligned} \tag{2}$$

Where $\gamma$ is the total emission rate that together with the pure dephasing rate $\gamma_{\mathrm{dp}}$ constitutes $\gamma_2 = \gamma/2 + \gamma_{\mathrm{dp}}$. While the population is also dependent on the driving field amplitude through Rabi frequency $\Omega = \mathbf{d}\cdot\mathbf{E}/\hbar$.

In a single-mode conventional waveguide, the resulting transmitted "output" electric field can be expressed in terms of the input driving field[1, 2]:

$$\hat{\mathbf{E}}^+_{\mathrm{out}}(\mathbf{r}) = \hat{\mathbf{E}}^+_{\mathrm{in}}(\mathbf{r}) + i\frac{\beta\gamma}{2\Omega}\hat{\mathbf{E}}^+_{\mathrm{in}}(\mathbf{r})\hat{\sigma}_{ge} \tag{3}$$

Where waveguide-emitter coupling efficiency is governed by the ratio $\beta = \frac{\gamma_{\mathrm{WG}}}{\gamma}$. Here $\gamma_{\mathrm{WG}}$ is the rate of decay into the waveguide mode. The coupling factor is divided by 2 as equal coupling to both directions of propagation is assumed i.e. the coupling is isotropic. From this we define the corresponding transmission coefficient $t$ that transforms the input electric field $\hat{\mathbf{E}}^+_{\mathrm{in}}(\mathbf{r})$ to $\hat{\mathbf{E}}^+_{\mathrm{out}}(\mathbf{r})$ through the photonic waveguide. Using equation 3, results in:

$$t = \frac{\langle\hat{\mathbf{E}}^+_{\mathrm{out}}(\mathbf{r})\rangle_{\mathrm{ss}}}{\langle\hat{\mathbf{E}}^+_{\mathrm{in}}(\mathbf{r})\rangle_{\mathrm{ss}}} = 1 + i\frac{\beta\gamma}{2\Omega}\rho_{eg} \tag{4}$$

Inserting the density matrix element $\rho_{eg} = \rho^*_{ge}$ of equation (2), we obtain:

$$t = 1 - \frac{\beta\gamma}{2}\frac{(\gamma_2 + i\Delta)}{\gamma_2^2 + \Delta^2 + 4(\gamma_2/\gamma)\Omega^2} \tag{5}$$

Finally, the normalized intensity of the transmitted light can be calculated as:

$$\begin{aligned}I_t &= \frac{\langle\hat{\mathbf{E}}^-_{\mathrm{out}}(\mathbf{r})\hat{\mathbf{E}}^+_{\mathrm{out}}(\mathbf{r})\rangle_{\mathrm{ss}}}{\langle\hat{\mathbf{E}}^-_{\mathrm{in}}(\mathbf{r})\hat{\mathbf{E}}^+_{\mathrm{in}}(\mathbf{r})\rangle_{\mathrm{ss}}}\\ &= 1 - \frac{\beta\gamma\gamma_2(2-\beta)}{2(\gamma_2^2 + \Delta^2 + 4(\gamma_2/\gamma)\Omega^2)}\end{aligned} \tag{6}$$

We emphasize that $I_t \neq |t|^2$.

### A. Maximal phase shift

The maxima of the phase shift with respect to the detuning can be found, at low power ($\Omega \ll 1$) and in the absence of dephasing, by solving:

$$\frac{\partial\arg(t)}{\partial\Delta}(\Delta_\pm) = \frac{2\beta\gamma\left((\beta-1)\gamma^2 + 4\Delta_\pm^2\right)}{\left(\gamma^2 + 4\Delta_\pm^2\right)\left((\beta-1)^2\gamma^2 + 4\Delta_\pm^2\right)} = 0 \tag{7}$$

which corresponds to

$$\Delta_\pm = \pm\gamma\frac{\sqrt{1-\beta}}{2} \tag{8}$$

Plugging this back in the expression of the argument, one can find :

$$\begin{aligned}|\phi|_{\max} &= |\arg(t(\Delta_\pm)|\\ &= \arg\left(\frac{\left(2 - i\sqrt{1-\beta}\right)\beta - 2}{\beta - 2}\right)\\ &= \tan^{-1}\left(\frac{\beta}{2\sqrt{1-\beta}}\right)\end{aligned} \tag{9}$$

* lodahl@nbi.ku.dk

† hanna.le-jeannic@cnrs.fr

### B. Transmission for a chirally coupled emitter

In a waveguide with chiral light-matter coupling the interaction is directionally dependent. Similar to before, the total electric field in transmission is

$$\hat{\mathbf{E}}^+_{\text{out}}(\mathbf{r}) = \hat{\mathbf{E}}_{\text{in}}(\mathbf{r}) + i\frac{\beta_{\text{dir}}\gamma}{\Omega}\hat{\mathbf{E}}^+_{\text{in}}(\mathbf{r})\hat{\sigma}_{ge} \quad (10)$$

where we define the directional coupling efficiency as $\beta_{\text{dir}} = \gamma_t/\gamma$, by differing the emission rate in transmitted($t$) or reflected modes($r$). Following the same method as for conventional waveguide, we have:

$$\begin{aligned} t_{\text{dir}} &= 1 - \frac{\beta_{\text{dir}}\gamma(\gamma_2 + i\Delta)}{\gamma_2^2 + \Delta^2 + 4(\gamma_2/\gamma)\Omega^2} \\ I_{t_{\text{dir}}} &= 1 + \frac{2\beta_{\text{dir}}\gamma\gamma_2(\beta_{\text{dir}} - 1)}{\gamma_2^2 + \Delta^2 + 4(\gamma_2/\gamma)\Omega^2} \end{aligned} \quad (11)$$

Note that in the case of an isotropic, conventional waveguide ($\gamma_t = \gamma_r = \gamma_{\text{WG}}/2$), we recover the equation for an emitter coupled isotropically to waveguide modes.

## II. MACH-ZEHNDER INTERFEROMETRY

The intensity of the output modes in a Mach-Zehnder interferometer is affected by the difference in phase, $\delta\phi$, between the two paths in the interferometer:

$$I = \sin^2(\delta\phi/2) \quad (12)$$

When light at frequency $f$ travels through each arm of the Mach-Zender interferometer (1,2), it experiences a phase shift of $\phi_{1,2} = 2\pi f L_{1,2}/c n_{1,2}$, where the speed of light is c and the index of refraction $n$ may be different in the two arms with respective distances $L_{1,2}$. Additionally, there may be an environmental fluctuation phase difference $\delta\phi_{\text{env}}$. Only one path (path 1) is affected by a phase change $\phi_{\text{QD}} = \arg(t)$ induced by the quantum dot waveguide system. Therefore, the final interferometric phase difference can be expressed as

$$\delta\phi = \phi_1 - \phi_2 = \frac{2\pi f \delta L}{c} + \delta\phi_{\text{env}} + \phi_{\text{QD}} \quad (13)$$

$\delta L$ is the interferometric path length difference. The interferometric signal obtained when sweeping the laser detuning is displayed in Fig. 1(b). The Fourier transform(FFT) of these interferometric fringes is displayed in Fig. SM SM1. Using Equations 12 and 13, we identify the main frequency component of the Fourier transform as $f = \delta L/c$ and we estimate the full path length difference of our interferometer to be $\delta L \approx 2.78$m.

The interferometer was stabilized by mounting a mirror in one of the Mach-Zehnder arms on a piezoelectric transducer(PZT). This allowed upon the application of a voltage to modulate the optical path length difference and thus correct for any fluctuation or drift. This Mirror/PZT system was found to have its first frequency harmonic at around 4KHz. By modulating the PZT voltage at 3.1 KHz with a small amplitude, a lock-in amplifier was used to gain a signal that looks roughly like the first derivative of the interferometric signal. Using this as the error signal for feedback PID loop to lock at the zero value of the lock-in output resulted in a top-of-fringe locking[3]. FIG.SM2 show in blue the normalized fringes signal recorded by modulating the PZT with a ramp signal. In the recorded range, the signal shows that the mirror was displaced roughly 4 wavelengths. Compared to this in black is the top-of-fringe locked signal taken in a subsequent measurement.

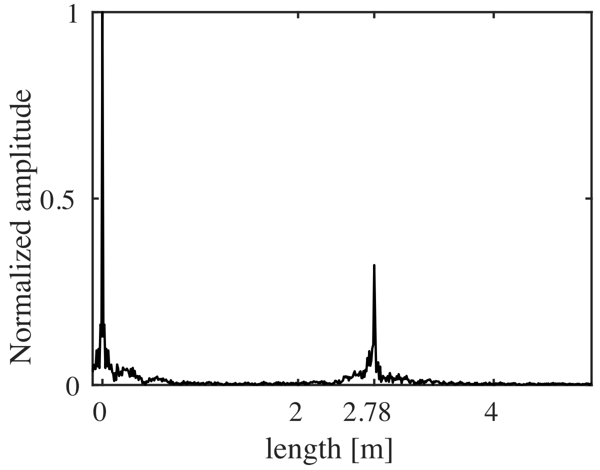


Fig. SM 1: Normalized Fourier transformation of the interferometric signal as function of $\delta L$ in meters with the QD turned off.

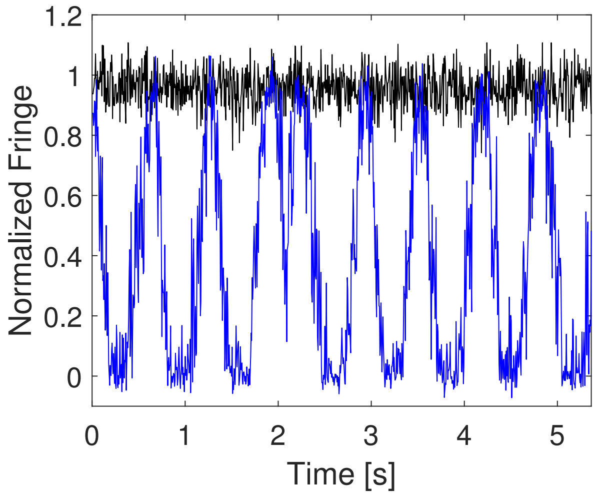


Fig. SM 2: Normalized and background subtracted fringe signal seen on the oscilloscope of the modulated interferometer(blue) and output of the stabilized interferometer (black)

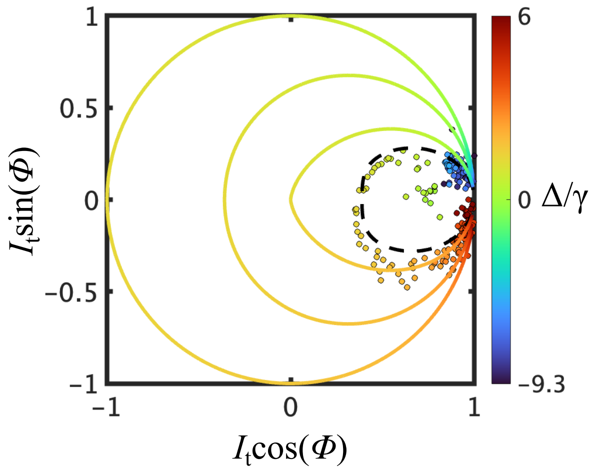


Fig. SM 3: Visualization of the effect of transition 2 (colored dots), and its corresponding fit (dashed black line) in a phasor diagram as a function of the normalized detuning $\Delta/\gamma$. For comparison, solid colored lines from inner to outer curves represent calculations for increasing directional coupling efficiencies $\beta_{\rm dir} = \{0.5, 0.8, 1\}$.

## III. MODELING THE EXPERIMENTAL DATA

### A. Phase and Intensity

We simultaneously fit the phase and intensity data of the two dipoles' response displayed in Fig. 2(c) and (d). We assume here for simplicity identical dephasing rates for both dipoles. Furthermore, we assume only pure dephasing, while in reality also slow noise processes (spectral diffusion) are influencing, however an unambiguous separation of these two processes is outside the scope of the present work; for more information, see [4]. As a consequence, the extracted pure dephasing rates will be overestimated. We adjusted the displayed data by taking into account the constant offset $\phi_0$ caused by weak Fano resonances, which are a result of partial reflection from the outcoupling gratings of the waveguide. (More information can be found in the references [4, 5]) We find the parameters to be:

| | Dipole 1 | Dipole 2 |
|---|---|---|
| $\beta$ | $0.94 \pm 0.03$ | 1 |
| $\gamma$ (ns$^{-1}$) | $9.4 \pm 0.2$ | $12.3 \pm 0.2.$ |
| $\gamma_{\rm dp}$ (ns$^{-1}$) | $3.9 \pm 0.1$ | |
| $\phi_0$ (rad) | $-0.25 \pm 0.02$ | |

We present the data of dipole transition 2, and the corresponding model fit in a phasor diagram in Fig. SM SM3

### B. Saturation Characterization

In the following, we focus only on transition (2). We fit all the transmission spectra series with power presented in Fig. 3 simultaneously using a nonlinear least-squares regression based on our model:

$$\phi(\Delta, \Omega) = \arg\left[1 - \frac{\beta\gamma(\gamma_2 + i\Delta)}{2\left(\Delta^2 + \gamma_2^2 + 4\Omega^2\gamma_2/\gamma\right)}\right] \qquad (14)$$

Since we do not know the exact Rabi frequency we define the mapping efficiency constant $\eta$ to the power as $\Omega = \sqrt{\eta P}$. We obtain the single set of parameters:

| | |
|---|---|
| $\beta$ | $0.99$ $[0.57, 1]$ |
| $\gamma$ (ns$^{-1}$) | $12.6$ $[7.7, 17.4]$ |
| $\gamma_{\rm dp}$ (ns$^{-1}$) | $3.4[0, 7.4]$ |
| $\phi_0$ (rad) | $-0.26$ $[-0.31, -0.2]$ |
| $\eta$ (rad.$s^{-1}$.mW$^{-1}$) | $5$ $[2.3, 7.7]$ |

Those values are in good agreement with the data of the two dipoles in Figure 2. At resonance, the saturation parameter is defined as $S = \frac{4\Omega^2}{\gamma\gamma_2}$ (where $\rho_{ee} = \frac{S}{2(S+1)} = 1/4$ at $S = 1$). By using the fitted parameters, this gives: $P_{\rm sat} = \frac{\gamma(\gamma/2+\gamma_{\rm dp})}{4\eta} \approx 0.15$ mW (indicated by a full line in Fig. 3(b)). Similarly, we can estimate the critical photon flux from the fitted parameters as: $n_c = \frac{1+2\beta\gamma_{\rm dp}/\gamma}{4\beta^2} \sim 0.39[0.25, 0.73]$ [4, 5]. This is comparable to previously measured values for solid states: quantum dots: $n_c \sim$ [0.81[5], 0.33[4], 1.6[6]] in weak coupling and $n_c \sim 0.4 - 0.6$ in strong coupling [7]; and Organic Molecules:$n_c \sim 25$[8] in weak coupling and $n_c \sim 0.44$[9] in strong coupling. An atom strongly coupled to a resonator has demonstrated even lower values, with $n_c \sim 0.02$[10]. At each power we also perform an independent fit with free parameters, to extract accurately the maximal, experimentally measured phase shift from the data. Those are the data points $|\phi_{\rm max}|$ displayed in Fig.3(b). By directly fitting by a general decay model $|\phi_{\rm max}| = Ae^{P/P^*_{\rm sat}}+B$, we find $P^*_{\rm sat} = 0.14$mW$[0.11, 0.16]$. This $P^*_{\rm sat}$ is the saturation power of the maximal phase shift, and is indicated by a dotted line in Fig. 3(b).

## IV. QD AND WAVEGUIDE SYSTEM

Initial experimental measurements consisted of investigating the transmission response of the photonic crystal waveguide and identifying an optical transition from a quantum dot embedded in the waeguide. A widefield image of a waveguide system leading through a photonic waveguide crystal is shown in FIG.SM4(a). Here the light can be coupled in/out of the waveguide via the couplers at the ends. The larger area along the waveguide is the photonic crystal consisting of a regular lattice with a periodicity of 250nm with hole sizes of 70nm. By collecting the transmitted signal we can obtain the transmission

response by scanning the laser frequency. The resulting transmission response is shown in FIG.SM4(b). The red and blue lines show respectively the laser frequency of the on-resonance laser and locking laser . When the band of the transmission was known, finer ranges of frequencies were scanned until the transmission dip of a quantum dot was found. By Scanning the frequency for different voltages we are able to build the transmission map as shown in FIG.SM5. The voltage for the experiment was set at 1.24V for the "on" mode i.e. we have turned on the optical transition. In contrast, the "off" mode had the voltage set to 0.8V sufficiently far from an optical transition.

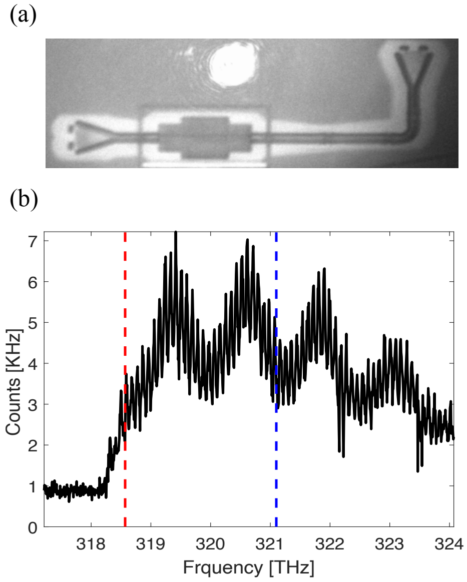


Fig. SM 4: (a) Widefield image of the sample with the photonic waveguide, with the laser spot next to it. (b) Unormalized transmission spectra of the laser as a function of the wavelength in the waveguide. The bandgap can be easily localized and is around 318.3 THz. The red and blue line marks the frequency of the on-resonance and locking laser, respectively.

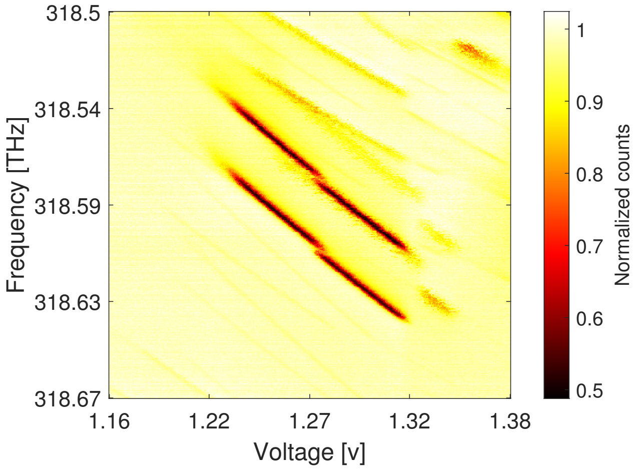


Fig. SM 5: Normalized transmission of the laser through the waveguide as function of the frequency and the voltage applied on the sample. The two dipole transitions can be identified.

[1] P. Türschmann, H. Le Jeannic, S. F. Simonsen, H. R. Haakh, S. Götzinger, V. Sandoghdar, P. Lodahl, and N. Rotenberg, Coherent nonlinear optics of quantum emitters in nanophotonic waveguides, Nanophotonics **8**, 1641 (2019).

[2] A. Asenjo-Garcia, J. D. Hood, D. E. Chang, and H. J. Kimble, Atom-light interactions in quasi-one-dimensional nanostructures: A green's-function perspective, Phys. Rev. A **95**, 033818 (2017).

[3] M. A. Luda, M. Drechsler, C. T. Schmiegelow, and J. Codnia, Compact embedded device for lock-in measurements and experiment active control, Review of Scientific Instruments **90**, 023106 (2019), https://doi.org/10.1063/1.5080345.

[4] H. Le Jeannic, T. Ramos, S. F. Simonsen, T. Pregnolato, Z. Liu, R. Schott, A. D. Wieck, A. Ludwig, N. Rotenberg, J. J. García-Ripoll, and P. Lodahl, Experimental reconstruction of the few-photon nonlinear scattering matrix from a single quantum dot in a nanophotonic waveguide, Phys. Rev. Lett. **126**, 023603 (2021).

[5] A. Javadi, I. Söllner, M. Arcari, S. L. Hansen, L. Midolo, S. Mahmoodian, G. Kiršanskė, T. Pregnolato, E. H. Lee, J. D. Song, S. Stobbe, and P. Lodahl, Single-photon non-linear optics with a quantum dot in a waveguide, Nat. Commun. **6**, 1 (2015).

[6] H. Thyrrestrup, G. Kirsanske, H. Le Jeannic, T. Pregnolato, L. Zhai, L. Raahauge, L. Midolo, N. Rotenberg, A. Javadi, R. Schott, A. D. Wieck, A. Ludwig, M. C. Löbl, I. Söllner, R. J. Warburton, and P. Lodahl, Quan-

tum optics with near-lifetime-limited quantum-dot transitions in a nanophotonic waveguide, Nano Letters **18**, 1801 (2018).

[7] I. Fushman, D. Englund, A. Faraon, N. Stoltz, P. Petroff, and J. Vučković, Controlled Phase Shifts with a Single Quantum Dot, Science **320**, 769 (2008).

[8] M. Pototschnig, Y. Chassagneux, J. Hwang, G. Zumofen, A. Renn, and V. Sandoghdar, Controlling the Phase of a Light Beam with a Single Molecule, Phys. Rev. Lett. **107**, 063001 (2011).

[9] D. Wang, H. Kelkar, D. Martin-Cano, D. Rattenbacher, A. Shkarin, T. Utikal, S. Götzinger, and V. Sandoghdar, Turning a molecule into a coherent two-level quantum system, Nat. Phys. **15**, 483 (2019).

[10] J. Volz, M. Scheucher, C. Junge, and A. Rauschenbeutel, Nonlinear $\pi$ phase shift for single fibre-guided photons interacting with a single resonator-enhanced atom, Nat. Photonics **8**, 965 (2014).

[H]
